# Pandemic as an Organizational Paradigm for Neonatal Care: Long-Term Impact of Mother–Infant Separation Practice During COVID-19

**DOI:** 10.3390/children12050592

**Published:** 2025-05-01

**Authors:** Maria Di Chiara, Benedetta De Santis, Flavia Gloria, Fabio Natale, Annarita Ferazzoli, Gianluigi Laccetta, Alessandra Marciano, Roberto Brunelli, Gianluca Terrin

**Affiliations:** Department of Mother and Child Health, Policlinico Umberto I, University “La Sapienza”, Viale del Policlinico 155, 00161 Rome, Italy; benedetta.desantis01@icatt.it (B.D.S.); gloria.1891102@studenti.uniroma1.it (F.G.); fab.natale@libero.it (F.N.); ferazzoli.1785678@studenti.uniroma1.it (A.F.); gianluigi.laccetta@uniroma1.it (G.L.); a.marciano@policlinicoumberto1.it (A.M.); roberto.brunelli@uniroma1.it (R.B.); gianluca.terrin@uniroma1.it (G.T.)

**Keywords:** COVID-19, breastfeeding, Rooming-In, growth, neonates

## Abstract

Objectives: The hospital organizational model can have an impact on people’s health. A critical lesson can be drawn from the pandemic. The possible negative sequelae of the practice of separation of maternal–infant dyads adopted during an infant’s first SARS-CoV-2 pandemic infection on infants have not been considered. Our purpose was to investigate the short- and long-term effects on neonates born to SARS-CoV-2 infected mothers of two different mother–infant dyad management strategies after birth (Separation vs. Rooming-In). Methods: This prospective cohort study enrolled 60 pregnant women who tested positive for SARS-CoV-2 infection and their newborns. We identified two cohorts of study based on mother–infant dyad management after delivery: Cohort A (Separation) and Cohort B (Rooming-In). Inclusion criteria were neonates born from mothers infected with SARS-CoV-2 during the pregnancy undergoing or not undergoing separation. Main Outcome: Rate of exclusive breastfeeding at 6 months of age was the primary outcome. The rate of mother–infant transmission of SARS-CoV-2 infection, growth, incidence of acute infections and neurodevelopment up to 12 months of life were also evaluated. Results: In total, 60 mother–infant dyads (maternal age 30.6 vs. 33.8 years, *p* = 0.335; gestational age 39.0 vs. 38.9 weeks, *p* = 0.451) were enrolled at delivery, and 53 dyads completed the study at the 6-month follow-up. Baseline clinical characteristics were similar between the two cohorts. At 6-month follow-up, the rate of breastfeeding was significantly decreased in Cohort A compared with Cohort B (4% vs. 46%, *p* < 0.001). The rate of SARS-CoV-2 infection was similar between the two cohorts of the study. Weight gain at 6 months of life was significantly higher in Cohort A compared to Cohort B (8129 g, 95% CI, 7562 to 8695; vs. 7393 g, 95% CI, 6912 to 7874; *p* = 0.005). No differences were detected in terms of rate of acute neonatal infections and neurodevelopment outcomes. Conclusions: The separation practice led to a reduction in the rate of breastfeeding after discharge and to a consequently increased implementation of formula milk, which might justify the alarming increased weight gain of newborns who did not undergo the Rooming-In practice. Given the potential of recurrent outbreaks of other viral pandemics, our results suggest more caution early in life towards the disruption of consolidated procedures that may have long-term consequences. However, the COVID-19 pandemic offered a unique context to observe the effects of temporary mother–infant separation; clinicians should be reassured that the temporary separation practice did not affect neurodevelopment and be aware that it could be considered an option, at least if Rooming-In cannot be carried out due to severe reasons such as lack of staff or adequate space.

## 1. Introduction

The hospital organizational model can have an impact on people’s health. Hospitals worldwide operate on tight financial margins with complex processes to match the overload of patients that can happen during a short-term crisis such as the severe acute respiratory syndrome coronavirus 2 (SARS-CoV-2) pandemic or, ultimately, a seasonal influenza outbreak [1,2]. The management of mother–infant dyads with newborns being near-term or term during the initial SARS-CoV-2 pandemic constituted a challenging issue for obstetricians and neonatologists. Given the scarce evidence regarding either pre-/perinatal or postnatal transmission from mother to child, conflicting recommendations targeting the management of mother–infant dyads were provided, particularly in the early stages of the pandemic [3]. Some health authorities and professional societies promoted Rooming-In even for mothers with SARS-CoV-2 infection at delivery [4,5,6,7], while others questioned the safety of mother–infant closeness [8,9]. Long-term outcomes beyond the neonatal period, as well as data on the consequences of initial management recommendations remain limited. A recent meta-analysis of uncontrolled studies examining infection rates in infants born to mothers with SARS-CoV-2 found an overall infection rate of SARS-CoV-2 of 2.3%, without significant differences between studies including neonates based on whether or not neonates were roomed-in with their mothers [10]. Even though the risk of neonatal infection has been demonstrated not to be affected by these mother–infant dyad practices, it has been reported that practicing separation can negatively affect the rate of breastfeeding during the hospital stay and at discharge [11,12]. Furthermore, we have previously demonstrated that mothers infected during the peripartum period confer protection to their newborns through breastmilk, delivering secretory-specific IgA against SARS-CoV-2 and actively stimulating and training the neonatal immune system development via breastmilk immune complexes [13]. It has been thoroughly studied that there are several interventions, very early in neonatal life, that can have an impact later in life. However, data reporting the effects of early mother–infant separation during the first SARS-CoV-2 pandemic on lasting neonatal outcomes have not been provided. The clinical management policy in our neonatal intensive care unit (NICU) changed over the pandemic period: at the beginning of the spread of the coronavirus, our policy was to separate mother and child after delivery. As soon as there emerged mounting evidence that Rooming-In was a safe approach, we decided to change our local practice. During the pandemic, it was necessary to implement use organizational models regardless of peer-reviewed evidence. Although this approach was justified by the emergency of the situation, we aimed to critically analyze the consequences. We hypothesized that early separation of mother and newborn in the immediate postnatal period could have a detrimental effect on the child’s health. The purpose of this prospective study was to compare the short- and long-term outcomes of neonates born to SARS-CoV-2-infected mothers under two different models of mother–infant care: Rooming-In versus separation.

## 2. Materials and Methods

We designed a prospective observational study during the COVID-19 pandemic, when Policlinico Umberto I Hospital of Rome was designated a regional reference center for pregnant women who tested positive for SARS-CoV-2 at delivery. We considered eligible all neonates with gestational age ≥ 35 weeks and birth weight > 1800 g, born from mothers positive for SARS-CoV-2 at delivery, consecutively observed in the NICU of Policlinico Umberto I Hospital of Rome between March 2020 and May 2023. We enrolled newborns meeting the criteria for Rooming-In including clinical stability at birth, absence of congenital malformation or infections other than SARS-CoV-2, and mother consent. Exclusion criteria were need for respiratory support or supplemental oxygen, vital signs out of the reference ranges, inability of the mother to take care of the baby, inborn errors of metabolism and death within 24 h of life. In our institution, during the pandemic the management of mothers with SARS-CoV-2 infection and their newborns have changed: from 1 March 2020, to the end of June 2022, infected mothers were separated from their newborns immediately after birth and were kept separated during the entire neonatal hospitalization.

Starting from July 2022, the practice of Rooming-In was re-adopted, returning to practices aligned with pre-pandemic standards. Thus, neonates were placed on their mother’s chest, immediately after birth and then placed in the same room for the whole length of the hospital stay. For the both cohorts the duration of separation refers to the time from birth until hospital discharge. In our setting, neonates are usually discharged approximately 72 h after birth. Breastfeeding was recommended by the staff during hospitalization and at discharge. Lactation support including education on hand expression, frequency of pumping, and arrangement of breast pumps was provided for isolated mothers intending to breastfeed. If breastfeeding was contraindicated or not possible, they were given formula 1, the same for all newborns. To verify the effects of this change in practice, we compared 2 temporal Cohorts: Cohort A (Separation) vs. Cohort B (Rooming-In). In our policy all subjects born from mothers positive for SARS-CoV-2 underwent a clinical follow up of 2 years.

### 2.1. Outcome Measures

We considered as primary outcome exclusive breastfeeding rate (maternal breast milk—either directly at the breast or expressed—without the addition of formula milk) at 6 months of age. Our secondary outcomes were duration of hospital stay, the incidence of main children’s infections and growth during the first 6 months of life. We also evaluated neurodevelopment at 12 months of life. We calculated a minimum sample size of 30 patients for each group of the study (2-sample *t*-test, 80% of power in hypothesis test, 0.05 of type 1 error, 2-tailed test, drop out 10%) to demonstrate a difference of about 30% in the rate of exclusive breastfeeding at 6 months of life between the two study cohorts. Thus, we recruited subjects of cohort A back in time and newborns of cohort B until the estimated sample size was reached. Subjects of cohort B were enrolled prospectively starting from July 2022. Two dyads were excluded because of lack of clinical data.

A post-hoc power analysis was conducted for the primary outcome using an alpha level of 0.05. The analysis indicated a statistical power of 89.6%, suggesting that the sample size was sufficient to detect significant differences for this endpoint.

### 2.2. Data Collection

Investigators who were not involved in the eligibility and enrollment phases, prospectively recorded obstetrics data including symptoms related to SARS-CoV-2 infection, prenatal, perinatal and postnatal data using a structured data form, form birth until discharge or transfer to another hospital.

All enrolled infants were included in a follow-up program with evaluation at 6 (±30 days) months of age. Physicians collected data regarding breast milk and introduction of complementary feeding, growth parameters and main infants’ clinical conditions and housing conditions they lived in.

At 12 months neurodevelopment was assessed by administering the Developmental Profile 3 (DP-3) by follow-up call interviews conducted by two physicians with expertise in neurodevelopmental follow-up [14,15].

### 2.3. Statistical Analysis

Statistical analysis was performed using the IBM Statistical Package for Social Science software (SPSS Inc., version 27.0 Chicago, IL, USA). We checked for normality using a Shapiro–Wilk test. The median and minimum-maximum range summarized continuous variables. Qualitative variables were expressed as number and percentage. We used an χ^2^ test or exact test for categorical variables and *t*-test, Mann–Whitney and Wilcoxon tests for paired and unpaired variables. A multivariate analysis was performed to evaluate the influence of covariates (cohort assignment, respiratory distress, feeding intolerance) on duration of hospital stay. We also performed a multivariate analysis to examine the influence of covariates (maternal age higher than 30 years old, firstborn infant, cohort assignment) on exclusive breastfeeding rate at 6 months. The level of significance for all statistical tests was two sided (*p* < 0.05). Data were elaborated by a statistician unaware of the study aims.

### 2.4. Ethics

The study was conducted in conformity with the World Medical Association Declaration of Helsinki24for medical research involving human participants and was approved by the Ethical Committee of Policlinico Umberto I in Rome, Italy.

## 3. Results

Demographic and clinical characteristics of the mothers and their newborns enrolled in the study, during hospital stay, are shown in Table 1. No statistical differences were found between the two study cohorts among baseline characteristics (Table 1).

**Figure 1:**
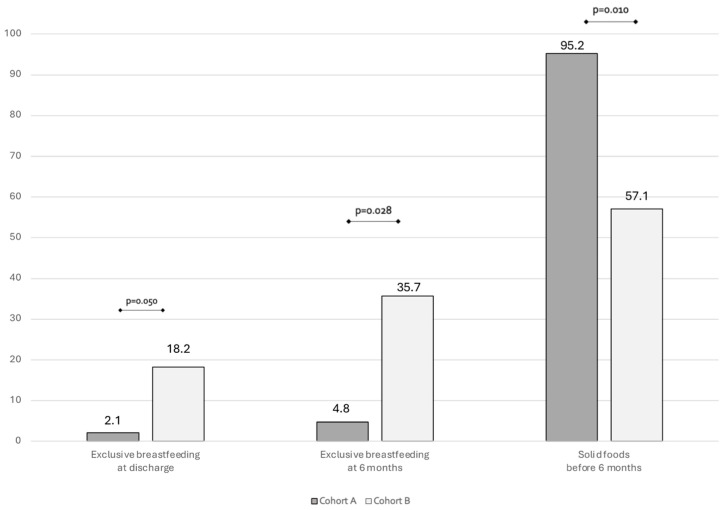
Bar plots showing the rate of breastfeeding and complementary foods at 6 months of life.

In Figure 1 we showed the type of feeding at 6 months follow-up. At 6 months follow up, a significant difference can be found in exclusive breastfeeding rate. Also, a significantly higher rate of introduction of solid foods before 6 months was found in Cohort A compared to Cohort B (Figure 1).

Table 2 presents the findings of a binary logistic regression analysis, performed to evaluate the influence of covariates on exclusive breastfeeding rate at 6 months. The results show cohort assignment influences exclusive breastfeeding rate at 6 months independently from maternal age and firstborn child (Table 2).

Length of hospital stay was found to be significantly higher in Cohort A compared to Cohort B (9.7 days; CI 95%, 5.4 to 14.4; vs. 5.3 days; CI 95%, 4.2 to 6.4; *p* = 0.005). We demonstrated that length of hospital stay depends on cohort assignment and not on perinatal factors in a binary logistic regression analysis (Supplementary Table S1).

In our population, SARS-CoV-2 infection rate was comparable among the two study cohorts both up to 28 days (6.7% vs. 6.7%, *p* = 0.694) and to 6 months (6.7% vs. 3.3%, *p* = 0.500). Rate of clinical conditions and housing features in the first 6 months of life were similar in Cohort A compared to Cohort B (Supplementary Table S3).

Growth parameters are shown in Table 3.

While length and head circumference are similar in the two cohorts, we found a significantly higher weight in Cohort A compared to Cohort B at 6 months of life (Table 3). Rooming-in was confirmed to significantly influence body weight at 6 months of life (Supplementary Table S2). No differences between the two cohorts were reported about the neurodevelopment up to 12 months of life, neither in any of the five domains (Physical, Adaptive behavior, Social-emotional, Cognitive, Communication) or in the General Development score, as shown in Table 4.

## 4. Discussion

The sudden adoption of a new organizational model consisting in a temporary maternal-infant physical separation of mothers and newborns to protect newborns from acquiring SARS-CoV-2 infection from mothers with COVID-19 at the time of delivery, affected the health of both mothers and their infants, In a controlled prospective study, we demonstrated that newborns of mother infected with SARS-CoV-2 during the pregnancy undergoing separate care immediately after birth experienced reduced breastfeeding rates during the first months of life. Based on our results, the disruption of Rooming-In practice seems to lead to increased length of hospitalization. Besides, these negative effects on the mother-infants’ dyads did not come along with a significant reduction of neonatal risk infection, during perinatal period. Long-term findings also revealed an increased weight gain, in the first months of life, in babies who underwent separate care at birth, likely due to either the increased use of formula milk or complementary food in these subjects. Finally, our data help to alleviate concerns about the consequences of disrupted Rooming-In on neurodevelopment. Rooming-In practice seems to support breastfeeding continuation up to six months of age. However, deviation from care practices known to enhance mother-infant interaction has significantly lowered breastfeeding rates at discharge [14]. Current literature also highlights that the duration of exclusive breastfeeding, globally, remains low warranting the need to reinforce of care practices proven to be effective [15,16]. Previous studies have investigated the effects of separation practice during SARS-CoV-2 pandemic on breast milk. A retrospective cohort study, based on an anonymous worldwide online survey demonstrated that infants who did not room-in within were significantly less likely to be exclusively breastfed in the first 3 months of live. In this study participating people were recruited through social media, support groups, and health care providers, during the first wave of pandemic [17]. Previously, Rostomian et al. in a longitudinal follow-up of 33 publicly insured infants born to mothers with SARS-CoV2 infection in pregnancy showed a lower rate of breastfeeding in case of mother-infant separation [18]. They demonstrated that mother-infant separation was associated with adverse effects on breast milk feeding outcomes assessed up to 1-month post-discharge, but this change was not sustained at 2 months subsequent visits [18]. However, no data on breastfeeding was provided at 6 months of life, the sample size was small, and findings on developmental outcome was incomplete. In addition, this study was only conducted during the initial wave of COVID-19 cases in the United States. Only few studies compared the impact of separation vs. no separation at birth on breastfeeding. However, those studies have investigated the rate of exclusive breastfeeding up to a maximum of 3 months of life; none of them has carried a follow up investigation until the age of 6 months of life [19,20]. Popofsky et al. in a longitudinal observational study found a statistically significant lower rate of breastfeeding among separated dyads, compared to unseparated dyads. Nevertheless, follow up survey had a mean duration of 45 days of life [19]. Costa et al., in a retrospective cohort study, compared the rate of exclusive breastfeeding in a population of COVID-19 infected mothers and their newborn infants, either roomed-in or subject to separation practice. Costa reported a significantly higher rate of exclusive breastfeeding in the Rooming-In group, although the results were not corrected for confounding variables [20].

In keeping with previous evidence, we observed a low rate of SARS-CoV-2 infection in babies born from mothers with COVID-19, without significant differences between newborns who were separated from their mothers and those who were [21]. In other words, This suggests that avoiding the well-established benefits of Rooming-In practice is not justified by the aim of reducing neonatal infection risk [22]. Finally, we demonstrated in a multivariate model that separation at birth independently increases the duration of hospital stay regardless of perinatal complications such as respiratory distress and feeding intolerance. The extension of hospitalization, most likely may have a direct role on the rate of breastfeeding at discharge.

Previous observational studies have investigated whether neonatal exposure to SARS-CoV-2 infection may affect the risk of developing clinical conditions in during the first months of life [21,23]. Those studies drawn up that SARS-CoV-2 infection does not increase the risk of occurrence of major clinical conditions in the first months of life [21,23]. However, whether either one the two the potential impact of different mother-infant dyads management approaches on babies‘ long-term clinical conditions has not been previously evaluated. In our study, we also detected infants’ clinical conditions occurring during the first 6 months of life. Our results suggest that separation practice management seems to not affect the rate of children main clinical conditions, during the first months of life. To our knowledge, this is the first study aiming to detect the difference in the rate of the predominant infectious clinical conditions, up to 6 months of life, between two groups of neonates which differed from the mother-infant dyads management at birth.

The observed reduction in the rate of breastfeeding during the first months of life was associated with increased body weight at 6 months of life in babies who were separated at birth from their SARS-CoV-2 infected mothers. Over recent decades, there has been extensive interest in infant-feeding factors which could increase the risk of developing overweight/obesity during infancy, with particular emphasis on the differences between formula feeding and breastfeeding [24,25]. It has been demonstrated that infants predominantly fed with breast milk more than infant formula, or who were breastfed for longer periods, exhibit a lower risk of developing overweight in later childhood and adolescence [24]. The increase in body weight, without a corresponding increase in length, observed among separated infants, may be a concerning consequence of the interruption of the Rooming-In practice during the first phase of the pandemic. Possible alterations of body composition in formula-fed infants could be due to a variety of factors, including inconsistent caregiver responsiveness to infant cues such as recognition of hunger and satiety signals, as well as to macronutrient composition of formula feeds [26,27,28]. Further studies are needed to determine whether these findings, on the effect of the separation of dyads at birth during the pandemic on body weight at 6 months of age, persist throughout childhood. Long-term data on the neurodevelopment outcomes of infants born to mothers with SARS-CoV-2 infection during pregnancy are currently lacking. Recently it has been suggested that birth during the pandemic, irrespective of SARS-CoV-2 infection status, may be associated with altered neurodevelopment trajectories later in life [29]. Notably, no data are available comparing the long-term neurodevelopment of infants, born during the first pandemic wave to mothers with SARS-CoV-2 infection, who were separated at birth versus those who have experienced Rooming-In. Recently, Wu et al. reported data of a prospective observational cohort study, performed in China during the first wave of the pandemic (from 1 May to 31 October 2020) [29]. This study, enrolling a limited number of mother-infant dyads, demonstrated by a multivariable linear regression model, that the length of mother–infant separation was negatively correlated with the gross motor score at 3 months of life. Whether these effects persist throughout the lifespan remains controversial [29,30,31]. In our study, comparing infants separated at birth with those not separated, we observed similar neurodevelopmental outcomes at 12 months of life. However, our study was not designed to evaluate neurodevelopment outcome at 12 months of life, indeed, we found a high dropout rate which limits the impact and generalizability of our findings. Further studies, specifically designed on this outcome are advocated to better address this aspect.

The main strength of our study is that to our knowledge, this is the first controlled prospective design. Among the studies focusing on different managements of mother-infant pairs in case of SARS-CoV-2 maternal infection, this is the first one including a long-term follow-up, complete of clinical assessment, measurement of growth parameters, and evaluation of neurobehavioral development. Despite the interesting findings, the results of this study should be interpreted considering some limitations. Our findings may be related to the effects of chance (random error), bias, or confounding factors. To limit this bias, we verified whether the effects on the main outcomes were influenced by confounding variables. Despite our efforts, unknown confounding variables or those not considered in our statistical analysis may have influenced the study results. This is not a randomized clinical trial (RCT) because a blinded RCT on this matter would not be ethically feasible. The study was not specifically powered to detect differences in non-primary outcomes. Given the pragmatic design and the limited sample size, the possibility of type II error cannot be excluded for secondary outcomes. To limit bias, neonatologists taking care of the babies were unaware of the aims of our study; researchers who collected data for the statistical analysis were not involved in clinical practice and were unaware of the cohort assignment. The observed rate of SARS-CoV-2 infection refers only to confirmed ones, based on positive diagnostic testing. We fully acknowledge that this may not reflect the actual, full rate of infection, especially given the high proportion of asymptomatic cases in neonates. The follow-up drop out is elevated. It is likely that, following the pandemic, the patients were restless and did not want to participate in the study. Despite no other changes in the policies of care during the study period and the similar baseline characteristics of the two cohorts, it is not possible to exclude the chance that unknown differences in the clinical practice or changes in the medical staff composition may have influenced the results. Finally, all these considerations should take into account that since the first pandemic wave there have been numerous variants of SARS-CoV-2, each with its own characteristics of virulence and contagiousness. In a further step, it will be worth understanding how the emergence of highly contagious variants may also have affected the outcomes measured on this study. The assessment of attachment, mother infant interaction, postpartum maternal wellbeing were not co-assessed. In this study, we focused on aspects related to mother-infant separation, its impact on breastfeeding, and long-term outcomes. However, considering the historical context of the pandemic, we deliberately chose not to further burden patients and healthcare professionals during such a critical and sensitive period.

## 5. Conclusions

The findings of this study provide evidence-based information for the management of mother-infant dyads in cases of SARS-CoV-2 maternal infection, suggesting that Rooming-In can be safely adopted in order to improve breastfeeding in the first months of life. Separation of dyads at birth was found to be unhelpful in reducing the risk of mother-to-infant transmission, while it led to an increased and unjustified prolongation of hospitalization and disruption of consolidated breastfeeding practices, which in turn, has had, albeit indirectly, a negative impact on long-term body composition. More caution is needed, early in life, in implementing early-life procedures and interventions that may have unintended long-term consequences.

While it is true that changing well-established protocols such as Rooming-In without solid evidence can be risky, the COVID-19 pandemic offered a unique context to observe the effects of temporary mother-infant separation. Our results suggest that, although such separation is no longer acceptable in light of current evidence and guidelines, it did not result in negative effects on neurodevelopment; therefore, separation at birth could be considered an option, solely if rooming-in cannot be carried out due to severe reasons such as lack of staff or adequate spaces.

Going forward, our results can be worthful for improving the management of future health emergencies or any other form of overload for national health systems.

## Figures and Tables

**Table 1:** Demographic and clinical characteristics of mothers and neonates enrolled in the study.

	Cohort A Separation	Cohort B Rooming-In	*p* Value
Mother-infants’ dyads	n = 30	n = 30	
Maternal age at delivery	33.2 (30.8 to 35.5)	33.3 (30.8 to 35.8)	0.964
Native Italian, No. (%)	19 (63.3)	16 (53.3)	0.517
Gestational diabetes, No. (%)	7 (23.3)	3 (10.0)	0.114
Thyroid disorders, No. (%)	1 (3.3)	4 (13.3)	0.238
Urinary or genital tract infection, No. (%)	1 (3.3)	1 (3.3)	1.000
Intrauterine growth restriction, No. (%)	0 (0.0)	0 (0.0)	NA
Antibiotic prophylaxis, No. (%)	8 (26.7)	10 (33.3)	0.846
Twin pregnancy, No. (%)	0 (0.0)	0 (0.0)	NA
Antenatal corticosteroids, No. (%)	0 (0.0)	0 (0.0)	NA
Caesarean section, No. (%)	14 (48.3)	15 (50.0)	0.591
**Symptoms related to SARS-CoV2 infection of mothers**			
Symptomatic infection, No. (%)	18 (60.0)	15 (50.0)	0.459
Fever, No. (%)	11 (36.7)	6 (20.0)	0.238
Cough, rhinitis, or sore throat, No. (%)	13 (43.3)	12 (40.0)	0.480
Ageusia or dysgeusia, No. (%)	0 (0.0)	1 (3.3)	0.273
Myalgia or arthralgia, No. (%)	3 (10.0)	1 (3.3)	0.316
Headache, No. (%)	3 (10.0)	1 (3.3)	0.316
Chest pain or dyspnea, No. (%)	1 (3.3)	0 (0.0)	0.307
Hospitalization due to COVID-19	1 (3.3)	0 (0.0)	0.307
**Neonatal features**			
Female sex, No. (%)	11 (36.7)	13 (43.3)	0.396
Gestational age, weeks	38.6 (37.9 to 39.3)	38.8 (38.4 to 39.2)	0.617
Inborn, No. (%)	26 (86.7)	28 (93.3)	0.584
Firstborn, No. (%)	13 (43.3)	12 (40.0)	0.305
Resuscitation at birth, No. (%)	1 (3.3)	1 (3.3)	1.000
Birth weight, g	3367 (3202 to 3531)	3325 (3181 to 3470)	0.703
Birth length, cm	49.0 (48.1 to 49.8)	49.45 (48.5 to 50.3)	0.449
	**Cohort A** Separation	**Cohort B** Rooming-In	***p* v** **alue**
Birth head circumference, cm	33.9 (33.1 to 34.7)	34.1 (33.7 to 34.5)	0.612
5-min APGAR score	9.5 (9.3 to 9.8)	9.5(9.4 to 9.8)	1.000
pH on cord blood	7.24 (7.20 to 7.28)	7.26 (7.2 to 7.2)	0.671
Base excess on cord blood, mmol/L	−6.1 (−8.2 to −3.9)	−5.1 (−8.9 to −1.2)	0.612
**Neonatal clinical conditions during hospital stay**			
Respiratory distress, No. (%)	0 (0.0)	0 (0.0)	NA
Cough, rhinitis, or conjunctivitis, No. (%)	0 (0.0)	0 (0.0)	NA
Feeding intolerance, No. (%)	3 (10.0)	2 (6.7)	0.549
Diarrhoea, No. (%)	0 (0.0)	0 (0.0)	NA
Jaundice requiring phototherapy, No. (%)	4 (13.3)	2 (6.7)	0.689
Pneumonia, No. (%)	1 (3.3)	0 (0.0)	0.601
Antibiotic treatment, No. (%)	2 (6.7)	0 (0.0)	0.355

**Notes**. An intramuscular steroids cycle in two doses of 12 mg over a 24-h period; Data were expressed as mean (lower to upper limits 95% confidence interval), when not specified.

**Table 2:** Binary logistic regression model on exclusive breastfeeding rate at 6 months.

Variables	ß	S.E.	Wald	*p* Value	Odds Ratio (OR)	95 C.I for OR	
						Lower	Upper
Rooming-In	2.467	1.020	5.853	0.016 *	11.783	1.597	86.916
Maternal age	−2.025	1.142	3.144	*0.076*	0.132	0.014	1.238
Firstborn	−0.286	0.876	0.107	*0.744*	0.751	0.135	4.185
Native Italian	−0.698	0.942	0.548	*0.459*	0.498	0.078	3.156
Maternal symptomatic infection	−1.209	0.928	1.696	*0.193*	0.299	0.048	1.841

**Notes**. * *p* < 0.001.

**Table 3:** Growth parameters of newborns enrolled in the study, up to 6 months of life.

	Group 1	Group 2	*p* Value
*Unstandardized parameters*			
Weight, g	8129 (7562 to 8695)	7393 (6912 to 7874)	*0.005* *
Length, cm	69.2 (66.9 to 71.6)	67.9 (65.6 to 69.8)	0.298
Cranial circumference, cm	44.2 (41.6 to 46.8)	42.5 (40.0 to 44.9)	0.279
*Standardized Z-Score*			
Weight, g	0.5 (−0.3 to 0.6)	−0.2 (−0.8 to 0.4)	0.387
Length, cm	0.5 (−0.3 to 1.5)	0.8 (−0.2 to 1.7)	0.645
Cranial circumference, cm	0.5 (−1.1 to 2.3)	0.0 (−1.4 to 1.6)	0.649

**Notes**. Data were expressed as mean (lower to upper limits 95% confidence interval), when not specified. * *p* < 0.001.

**Table 4:** Neurodevelopment assessed by DP-3 at 12 months of age.

	Cohort A Separation	Cohort B Rooming-In	*p* Value
**Physical scale (M)**			
M standard	103.1 (96.8 to 109.39)	101.1 (96.7 to 105.5)	0.606
M centile	56.0 (43.15 to 68.8)	52.7 (43.2 to 62.2)	0.587
**Adaptive Behaviour scale (A)**			
A standard	100.2 (95.1 to 105.4)	97.6 (92.53 to 102.77)	0.464
A centile	50.8 (39.9 to 61.8)	43.5 (32.3 to 54.6)	0.338
**Social-emotional scale (S)**			
S standard	99.7 (94.9 to 104.6)	100.6 (94.5 to 106.62)	0.820
S centile	50.1 (39.6 to 60.6)	49.7 (37.5 to 61.8)	0.957
**Cognitive scale (G)**			
G standard	101.1 (95.5 to 106.8)	103.9 (98.6 to 109.2)	0.477
G centile	53.21 (42.2 to 64.3)	57.1 (46.2 to 68.0)	0.607
**Communication scale (C)**			
C standard	97.8 (93.0 to 102.7)	99.6 (93.7 to 105.5)	0.629
C centile	44.9 (35.7 to 58.0)	100.5 (34.8 to 54.9)	0.701
**General Development score**	100.5 (95.3 to 105.7)	100.4 (93.8 to 106.9)	0.984

**Notes**. Data were expressed as mean (lower to upper limits 95% confidence interval), when not specified.

## Data Availability

The datasets analyzed during the current study are available from the corresponding author on reasonable request.

## Supplementary Materials

The following supporting information can be downloaded at: https://www.mdpi.com/article/10.3390/children12050592/s1, Table S1: Binary logistic regression model on length of hospitalization; Table S2: Binary logistic regression model on growth at 6 months; Table S3: Clinical conditions and housing features at 6 months follow up.
