# Supplementary material for: Pandemic as an Organizational Paradigm for Neonatal Care: Long-Term Impact of Mother–Infant Separation Practice During COVID-19
Source: Children (Basel). 2025 May 1;12(5):592. doi: 10.3390/children12050592 (PMC12110108; doi:10.3390/children12050592)
Supplement: Supplementary file 1 [file children-12-00592-s001.zip › children-3540491-supplementary.pdf]

Supplementary Table S1. Binary logistic regression model on length of hospitalization.

Variables	ß	S.E.	Wald	*p value*	Odds ratio (OR)	95 C.I for OR	
						Lower	Upper
Rooming-in	-0.913	0.450	4.114	0.043*	0.401	0.166	0.970
Respiratory distress	-0.440	0.902	0.238	0.626	0.644	0.110	3.770
Feeding intolerance	1.368	1.112	1.514	0.219	3.927	0.444	34.718

Notes. *$p < 0.05$.

Supplementary Table S2. Binary logistic regression model on growth at 6 months.

Variables	ß	S.E.	Wald	*p value*	Odds ratio (OR)	95 C.I for OR	
						Lower	Upper
Rooming-in	-2.320	1.227	3.574	0.05*	0.098	0.009	1.089
Firstborn	0.664	0.823	0.650	0.420	1.943	0.387	9.758
Length of hospital stay [a]	1.360	1.077	1.596	0.206	3.897	0.472	32.160
Breastfeeding at 6 months	1.422	1.257	1.280	0.258	4.146	0.353	48.682

Notes. [a] Length of hospital stay more than 3 days.
*$p < 0.05$.

Supplementary Table S3. Clinical conditions and housing features at 6 months follow up.

	**Cohort A**<br>Separation<br>n= 26	**Cohort B**<br>Rooming-in<br>n= 28	***p value***
**Clinical features**			
Upper respiratory tract infections, No. (%)	13 (52.0)	10 (35.7)	0.094
Otitis, No. (%)	1 (4.0)	2 (7.1)	0.080
Bronchiolitis, No. (%)	5 (20.0)	2 (7.1)	0.060
Atopic dermatitis, No. (%)	0 (0.0)	3 (14.3)	0.094
Episode of vomit, No. (%)	0 (0.0)	1 (3.6)	0.060
Diarrhoea, No. (%)	2 (8.0)	2 (7.1)	0.102
**Housing conditions**			
Urban context, No. (%)	24 (96)	12 (42.8)	0.351
Cohabitants, *median (IQR)*	2.5 (2)	3.0 (1)	0.265
Pre-school aged cohabitants, *median (IQR)*	0.0 (0)	0.0 (0)	0.721
School aged cohabitants, *median (IQR)*	0.0 (1)	0.0 (1)	0.541
Any SARS-CoV-2 positive cohabitant, No. (%)	3 (12.0)	4 (14.3)	0.253

Notes. *$p < 0.05$.
